# Hypodermic Morphia

**Published:** 1882-12

**Authors:** 


Hypodermic Morphia.—One of the most eminent of German medical men is reported as saying that there probably are not less than ten thousand persons in Germany who have become slaves to the habit of hypodermically injecting morphine. There are many who take as much as eighteen injections every day. Some have hardly a square inch of skin on their bodies which is not marked by scars produced by this practice. Slaves of this habit are even more hopelessly enchained than those who take opium in other ways, and it is speedier destruction.—*Ext.*

